# Assimilation and ethnic marriage squeeze in early 20^th^ century America: A gender perspective

**DOI:** 10.4054/demres.2020.42.4

**Published:** 2020-01-14

**Authors:** Inbar Weiss, Guy Stecklov

**Affiliations:** 1 Department of Sociology and Population Research Center, University of Texas at Austin, Texas, USA; 2 Department of Sociology, The University of British Columbia, Canada

## Abstract

**BACKGROUND:**

During the 19^th^ and early 20^th^ centuries, large waves of international immigrants, often heterogeneous in terms of age and sex structure, arrived in the United States. Within a relatively short time, many of these immigrants were assimilated. While prior studies have identified an impact of the marriage squeeze on intermarriage, the role of gender is less known.

**METHODS:**

We use data from the 1930 census to examine the role played by variation in the sex ratios of the six largest immigrant groups at the beginning of the 20^th^ century on marital outcomes by sex.

**RESULTS:**

Our analyses show that the probability of marrying outside one’s ethnic group in this period is strongly tied to local ethnic sex ratios. Marital outcomes are affected for both sexes, but sex ratios are found to be more influential on males marrying out of their ethnic group. While a surplus of one’s own sex increases the probability of exogamy for males, it is likely to increase the probability of being single for females.

**CONTRIBUTION:**

Our findings highlight the importance of ethnic sex ratios in local marriage markets at a critical juncture of American immigration and its consequences. We focus on an understudied aspect of this process: gender differences in the association between sex ratios and marital assimilation. We show that marital decisions differed by sex and that the high levels of intermarriage in this period are more likely to be explained by unbalanced sex ratios for males than for females.

## 1. Introduction

Unprecedented waves of European migration to the United States from the late 19^th^ century through the early 20^th^ century not only helped to propel American progress (Hirschman 2005), but also generated the demographic structure that facilitated ethnic assimilation. Sociologists who have described the assimilation of the European immigrants in this and later periods highlight intermarriage as one of its fundamental driving forces (Alba and Nee 2003; Gordon 1964). Heterogeneity in ethnic sex ratios, generated by large-scale inflows of immigrants (Donato et al. 2011; Haines 2000; Warner and Srole 1945) played a key role in the increased number of ethnic-intermarriages (McCaa 1993; Pagnini and Morgan 1990) and helped in the assimilation of European immigrants in early 20^th^ century America.

The association between unbalanced ethnic sex ratios and intermarriage has been largely studied (Chiswick and Houseworth 2011; Graeme and Nguyen 2007; Hwang, Saenz, and Aguirre 1997), although gender differences in this relationship have received less attention. In addition to general differences in women and men’s behavior in the marriage market (Buss 1989), the uneven economic and legal resources that individuals were able to exchange in marriage and the two separate spheres ideology that characterized the early 20^th^ century (Figart, Mutari, and Power 2005) suggest that the effect of the marriage squeeze on assimilation might have differed by gender.

The goal of this paper is to shed light on gender variation in the impact of the ethnic marriage squeeze on marital assimilation among immigrants. To do so, we explore spatial variation in age and ethnic composition of the US population using data on the six largest immigration groups at the beginning of the 20^th^ century from the 1930 IPUMS census file (Ruggles et al. 2018). We define marital assimilation as a marriage between people of different ethnic backgrounds. In this paper, we use the terms marital assimilation, intermarriage, and exogamous marriages interchangeably.

The role of gender is explored through two additional subquestions. Do the sex ratios of *other* ethnic groups alter one’s own intermarriage prospects? And do ethnic sex ratios also affect nonmarriage – a viable competing alternative to exogamy? Our analysis includes multiple model specifications along with a series of alternative approaches to the measurement of sex ratios that help to ensure the robustness of our findings.

## 2. Background

Over the course of assimilation, individuals acquire elements and behavior of another culture as they are incorporated into larger groups (Castles, Haas, and Miller 2014; Park 1950). This is typically an intergenerational process caused by both conscious and unconscious actions of individuals and households (Alba and Nee 2003; Kraut 1990). As these processes unfold, there is an increase in the interaction between individuals from different backgrounds eventually leading to a blurring of the boundaries between groups (Waters and Gerstein Pineau 2016). The extent of the assimilation process depends on the characteristics of the different groups in the population and their willingness to accept one another (Richard 1991).

Intermarriage is a common indirect measure of both social integration (Waters and Gerstein Pineau 2016) and declining social intergroup distance (Qian and Lichter 2011). Gordon (1964) identified marital assimilation as one of the seven stages of assimilation. The growth of intermarriage (i.e., marital assimilation) is seen as a key outcome of structural assimilation, the large-scale entrance of a minority group into the social cliques and institutions of the majority. Intermarriage describes not just the willingness of the minority to assimilate, but also its acceptance by the majority (Blau 1977). Since assimilation is a process that starts with the arrival of the immigrant to the new society, groups and individuals that have been less exposed to the host society typically show lesser degrees of marital assimilation (Lieberson and Waters 1988; Schoen and Cohen 1980).

Blau, Beeker, and Fitzpatrick (1983) describe intermarriage as a precursor of assimilation – an act that resembles acceptance and the product of a process in which the boundaries between groups become less profound allowing individuals to commit to long-term unions (Alba 1995). In this sense, intermarriage is not just a union between individuals, but rather resembles a bridge between populations (Waters and Gerstein Pineau 2016). Immigrants are mostly assimilating to the native culture, although they might also be affecting this culture. Moreover, intermarriage shapes the social contexts of subsequent generations by unifying them under the continuously evolving mainstream culture (Alba and Nee 2003).

The increase of ethnic intermarriage among European immigrants during the first half of the 20^th^ century and the rapid accompanying assimilation offers a fertile ground for research on immigrant integration (Waters 1990). In the next section, we describe the characteristics of the European immigration and review the literature on the effect of sex ratios on their ethnic intermarriage. From this review, we develop our core arguments regarding the expected gender differences in this association. Next, we describe the data and analytical approach before presenting our results and interpreting our main findings. We end by discussing the implications and limitations of the study.

### 2.1 Massive migration flows and the salience of ethnicity in the early 20^th^ century

Ethnicity at the beginning of the 20^th^ century defined individual and group identity in a way that was tightly embedded in systems of stratification and segregation (Hirschman 1983). Americans classified themselves mainly by ethnic lines, their national origin or their nationality, and the American social structure was described as a series of subsocieties classified by ethnic identity (Gordon 1964). In this context, the ethnic community was central to the daily life of immigrants: It was at this level that many newspapers were published; colleges established; theaters and orchestras performed; holidays celebrated; workers’ unions and churches organized; and it served as a principle frame within which people worked and inhabited neighborhoods (Davis 1969; Furer 1973; Jacobson 2002; Juliani 1981; Lopata 1976; Renkiewicz 1973). While some immigrants were motivated or felt pressure to adopt new, more ‘American’ identities and behaviors (Goldstein and Stecklov 2016; Kraut 1990), others chose to focus more strongly on maintaining their culture and made efforts to protect their children from assimilating into American society (Alba and Nee 2003), in part by relying on ethnic networks that helped facilitate their own segregation (Gordon 1964).

During this period, unprecedented waves of immigrants arrived on America’s shores. Two aspects of this immigration era prove notable for helping to understand the changing context of ethnicity and marriage. First, immigrant flows from Southern and Eastern Europe were replacing the earlier North-West European migration streams. The “new” immigrants differed in their culture, religion and language and were more ethnically segregated than white natives and ‘old’ immigrants (Duncan and Lieberson 1959; Lieberson 1963; Waters 1990). In addition to the physical and social isolation that new immigrants usually experience (Lichter, Qian, and Tumin 2015), the hostility from natives and prior waves of immigrants toward the “new” immigrants (Waters 1990; Zinn 2014) also contributed to their delayed assimilation (Alba and Nee 2003; Pagnini and Morgan 1990).

The second contributing factor was more structural in nature: ethnic groups often arrived with dissimilar age and sex compositions that helped to destabilize local sex ratios (Guttentag and Secord 1983; Hirschman 2005; Warner and Srole 1945). International migrant flows caused a relative excess of males in the United States during this period, particularly among the foreign-born white population. In the 1910 census, for example, a ratio of 129 males for every 100 females was observed among foreign-born immigrants (Haines 2000). Though the immigrants were typically young, single males who came to search for new economic opportunities (Castles, Haas, and Miller 2014; Donato et al. 2011), there were also immigration streams dominated by young, single females. This was the case with the Irish immigration, which was strongly influenced by difficult marriage market conditions back in Ireland (Dixon 1978; Jackson 1984).

In addition to international migration flows, differential sex patterns of *domestic* migration across the United States could further accentuate or moderate spatial variation in ethnic sex ratios. Between 1920 and 1930, foreign-born domestic migration generated negative net migration rates in all the great plains states as a result of a movement toward the east and west coasts (Thornthwaite 1934). In addition, intensive urban growth, driven both by newly arrived international migrants as well as domestic migrants making their way to cities, helped to propel new demographic dynamics. America transitioned from a rural society with only 5% of citizens living in urban areas in 1790 to an urban society where more than a half of the population was urban by 1920 (Daniels 2002). Females, especially young ones, were more likely to migrate to the growing cities. In some cases, female migration balanced the sex ratios in cities, but in others it led to disproportionate share of women in urban areas and simultaneously created potential female deficits in many rural areas (Becker 1973).

### 2.2 Ethnic sex ratios and marriage

These underlying dynamics offer a compelling context to examine marital assimilation. On the one hand, the salience of ethnicity meant that marriage markets remained relatively closed with most marriages conducted within the boundaries of the ethnic group (Pagnini and Morgan 1990; Panunzio 1942; Wildsmith, Gutmann, and Gratton 2003). On the other hand, demographic processes could generate intense ethnic marriage squeezes, putting pressure on individuals and families to consider ethnic out-marriage.

A marriage squeeze is typically driven by a shortage of potential marital partners, and it has been shown to affect the age of marriage (Goldman, Westoff, and Hammerslough 1984; Schoen 1983), to alter the age gap between spouses (Bergstrom and Lam 1989; Muhsam 1974; Schoen 1983; Stier and Shavit 1994) and to affect the percentages that never marry, as well as divorce and remarriage rates (Angrist 2002; Goldman, Westoff, and Hammerslough 1984; South and Lloyd 1992). When populations are further distinguished by ethnicity, race, or religion, the marriage market squeeze can increase and in turn impact the levels of intermarriage, as members of the sex that are in surplus may become increasingly willing to expand their spousal search to other groups (Guttentag and Secord 1983).

Prior work has shed light on important consequences of the ethnic marriage squeeze on ethnic intermarriage in the early 20^th^ century. McCaa (1993) argued that the heavily skewed sex ratio in New York City at this period encouraged many to consider mates from different ethnicities and suggested that ethnic marital assimilation in the early stages of assimilation was driven primarily by extreme marriage squeezes. However, the gender gap in out-marriage was smaller than expected by the sex ratio, meaning that some immigrants who experienced the marriage squeeze decided (or were forced by the lack of potential partners) to remain single (McCaa 1993).

Spörlein, Schlueter, and van Tubergen (2014), taking a broader empirical perspective, estimated multilevel models on data consisting of over 140 ethnic groups in the United States over a 130-year period. Their analysis showed that exogamous marriages are associated with a shortage of ethnic spouses for males and females and that the instances of intermarriage became more frequent over time as the sex ratio become increasingly unbalanced. Wildsmith, Gutmann, and Gratton (2003) who also study trends in ethnic intermarriage since the late 19^th^ century, support the findings of the former and claim that the marriage squeeze’s effect on intermarriage weakened after WWII for immigrant groups who had arrived in the immediately preceding decades.

Most studies that focused on later periods or on other populations in the United States found broadly similar effects. For example, using the 1980 US census, Chiswick and Houseworth (2011) found that the likelihood of ethnic intermarriage decreased as the number of individuals from the opposite sex rose. Alongside new immigrants who arrived after 1965, their analysis also includes the offspring of the European immigrants, who are the focus of this paper.

### 2.3 Gender differences in the marriage market

Although gender differences in mate selection are well-known (Buss and Schmitt 1993; Feingold 1992; Sassler 2005; Sprecher, Sullivan, and Hatfield 1994), it is unclear whether ethnic sex ratios have comparable impacts on male and female ethnic exogamy patterns. We consider three factors that could have caused gender differences in the association between ethnic sex ratios and marital assimilation at the beginning of the 20^th^ century. First, economic theories of supply and demand suggest that when individuals experience a shortage of potential mates, they are forced to be more efficient and competitive in the marriage market and to be able to compensate partners from different ethnic groups in return for marriage (Angrist 2002; Guttentag and Secord 1983; Kalmijn 1998). In this sense, men usually have greater financial opportunities to offer in exchange for marriage than women (Becker 1973; Kalmijn 1998; Merton 1941). Empirical support for this underlying mechanism includes evidence that most of the exogamous marriages of Mexican-American women in Los Angeles in the 1960s were to high-status men (Mittelbach and Moore 1968) and similar results were found in New York among Puerto Rican women in the 1950s (Fitzpatrick 1966). Studies also found that a shortage of suitable men leads many women to stay unmarried rather than marry economically “unattractive” men (Crowder and Tolnay 2000; Lichter, Anderson, and Hayward 1995).

In contrast, women were less able to exchange resources in the marriage market in an era when most women weren’t financially independent. Although the job participation of young, single women increased in the early 20^th^ century, wage discrimination also increased and many labor market positions remained inaccessible to women (Goldin 1992). Moreover, separate spheres ideology was common at that time and working women were expected to leave their jobs after marriage. Thus, though 40% of single women were in the labor force, this number fell to only 6% amongst married women (Padavic and Reskin 2002). In practice, the two spheres ideology seriously reduced women’s attractiveness in the marriage market because single women’s beneficial jobs could not be leveraged in the marriage market mostly due to their temporal characteristics. Not only were women expected to leave their jobs upon marriage, but they were also the first to be laid off (Kessler-Harris 2001; McGuire 2008). Additionally, women who did remain in the labor force after marriage typically brought shame to the household (Padavic and Reskin 2002).

Second, legislation helped to further the interests of men and enabled them to marry out while punishing American women who married immigrant men. This is most evident in the 1855 statute that declared that foreign women marrying American men gained American citizenship, whereas American women marrying foreign men risked losing their own. Equal rights for exogamous marriage were only enacted in 1934 when Americans of both sexes gained the same naturalization benefits for their spouses (Cott 1998).

Third, the two spheres ideology had other implications on marriage. For men, work outside the house allowed them access and exposure to diverse populations. Women’s marital decisions have long been more tightly constrained by family preferences, particularly in this earlier era, and their exposure to outside groups was more carefully managed (Kerber 1988). Moreover, women were tied to more conventional marriage patterns and played a central role in transmitting ethnic identity to the next generation through food, holidays, and religious practices (Sassler 2005; Waters 1990). Minority women who were interested in transmitting their own ethnic culture to their children did it more efficiently in endogamous unions (Bisin and Verdier 2000).

To sum up, in contexts with shortages of suitable, marriageable men in the early 20^th^ century, women had to be willing to make more concessions by broadening the age range, socioeconomic status, and ethnic diversity of their potential mates to overcome the marriage squeeze.

### 2.4 Four hypotheses on ethnic sex ratios and exogamy

This study examines sex differences in the relation between ethnic sex ratios and ethnic intermarriage. Our starting point is to clarify a general effect of sex ratios on ethnic exogamy. We expect that shortages in the supply of potential partners from within one’s *own* ethnic group will propel out-marriage for both sexes. Since the sex ratio is typically measured as the ratio of males to females, we expect that sex ratios above 1.0 increase exogamy for males, and that sex ratios below 1.0 will increase exogamy for females.

Our second hypothesis considers whether ethnic sex ratios have similar impacts on the intermarriage patterns of males and females. Given women’s typically weaker economic and legal status in the marriage market and their more significant role in the socialization and acculturation of the next generation, we expect that the effect of unbalanced sex ratios on intermarriage will be stronger for males.

The effect of the ethnic sex ratios on out-marriage might also depend on the pool of potential mates from other ethnic groups in the same marriage market (markets defined by age and location). Thus, a shortage of same ethnic marriage opportunities may increase prospects through marriage to partners from a different ethnicity, but the feasibility of this alternative also depends on the sex ratio of the other ethnic groups in the same marriage market. To test this, we suggest a measurement of the sex ratios of other ethnic groups (OSR). Our third hypothesis is that the probability of exogamy will be higher for individuals when OSR are in their favor. Thus, a key modification introduced in our analysis is not only to consider the ethnic marriage market in one’s own ethnic group but also to base the exogamy analysis on the marriage market demography of other ethnic groups. Based on our earlier discussion, we expect that an increase in OSR will negatively affect males’ exogamy, as the pool of females from other ethnicities decreases. For females, we expect that an increase in OSR will have a positive but smaller effect on exogamy.

Lastly, most studies on marriage squeeze and marital assimilation ignore never-marrying as an additional outcome of extreme sex ratios. Groups may remain relatively homogenous either by purposefully avoiding out-marriage or by being shunned by other groups. Regardless, nonmarriage should be seen as a viable alternative to the marriage squeeze and as an outcome that can further decelerate the assimilation process. When comparing the two outcomes, we expect that the effect of unbalanced sex ratios will be stronger on nonmarriage than exogamy for both sexes. However, since women had fewer resources available to increase their odds of out-marriage and were more likely to remain unmarried in the face of a marriage squeeze, we expect to find a bigger gap between the two alternatives among females.

## 3. Method

### 3.1 Data

We analyze marriage patterns for over two and half million individuals using the full count Integrated Public Use Microdata Series (IPUMS) file for the 1930 US Census (Ruggles et al. 2018). In the 1930 census year, the number of foreign-born whites reached its peak, with almost 14 million white immigrants living in the United States (Haines 2000). This period concludes a massive wave of immigration from Europe and makes the 1930 census a compelling instrument for studying the relationship between sex ratios and marital assimilation. In addition, the 1930 census is preferable to the 1920 census, since the latter does not include a question on age at first marriage – an essential indicator we use to identify newly married individuals. In contrast, the 1930 census is preferable for our analysis over the 1940 census since the Immigration Act of 1924 sharply decreased the foreign-born population. In addition to sample size, the 1940 census is more likely to include first-generation immigrants with a higher average duration in the United States than the 1930 census, especially among immigrants from east and south Europe. The sample we use provides standard census information: sex, age, race, marital status, birthplace of both partners and their parents, household’s location (urban vs. rural), literacy, and the number of years in the United States for immigrants.

We restrict our analysis to white first and second-generation immigrants from the six largest emigration countries during this period: England, Germany, Ireland, Italy, Poland, and Russia. Non-Whites are excluded from this analysis since interracial marriages remained strongly sanctioned and, in some cases, legally restricted in 1930 (Cott 1998; Hollinger 2003; Rosenfeld 2008). Similarly, we try to restrict the sample to non-Jews, by excluding those whose mother tongue was Yiddish or Hebrew. In the early 20^th^ century inter-faith marriages between Jews and non-Jews were rare (Kennedy 1952). In addition, the distinctions among Jews from different ethnic origins were salient and until the 1970s some still considered a marriage between a Russian Jew and a German Jew an out-marriage (Waters 1990).

Three principal restrictions were imposed on the sample in the main analysis. Our sample of second-generation immigrants is limited to persons with two immigrant parents from the same country of origin or with one immigrant parent and one US-born parent. This restriction helped us avoid misidentification of the ethnic background of individuals, key to our categorization of ethnically endogamous and exogamous marriages.

Second, our analysis is restricted to marriage markets (in terms of ethnicity, age interval, and county) with a sex ratio between 0.11 to 9. This means our sample only contains individuals from ethnic marriage markets that were not hyper-restricted and contained at least 10% of both sexes. This step helps to reduce the effect of extremely atypical marriage markets. This restriction meant dropping 4.3% of cases for males and 0.6% for females (Table 1). We directly address the empirical consequences of restricting the boundaries of the marriage markets in a later robustness section.

Lastly, we restricted our sample to individuals who had never been married in 1930 and those newly married in the United States between 1929 and 1930. First-generation immigrants who married prior to arrival in the United States or married within a year of arrival were excluded. Overall, the age range of respondents is 23 to 53 years for males, and 20 to 50 for females. Our final sample consists of 2,639,591 observations, including 1,373,397 males and 1,264,347 females.

### 3.2 Methodology

Defining the boundaries of the *potential* marriage market is a critical step for measuring the role of ethnic sex ratios. The existing literature shows considerable variation in how ethnic sex ratios are measured: Sex ratios have been measured at the national (Angrist 2002; Goldman, Westoff, and Hammerslough 1984), state (Spörlein, Schlueter, and van Tubergen 2014; Wildsmith, Gutmann, and Gratton 2003), and metropolitan levels (Crowder and Tolnay 2000; Hwang, Saenz, and Aguirre 1997). These various choices are often driven by data limitations as much as theoretical arguments, but marriage markets, certainly in the early 1900s, are likely to be better operationalized using smaller geographical units since most mates were found locally (Akers 1967; Cox 1940; Ramsøy 1966). Our own analysis builds on the use of county-level data as the geographic unit for studying marriage markets in this period. This is consistent with prior evidence in support of both county and metropolitan levels as the preferable units to analyze the impact of sex ratios on marriage patterns (Fossett and Kiecolt 1991).

Our analysis considers both the demographic and ethnic constraints that individuals face in the marriage market. Exogamy, the dependent variable in our models, identifies whether an individual’s spouse belongs to an ethnic group different from one’s own. An individual’s ethnicity is defined either by country of origin for those who are foreign-born or by parental place of birth for second-generation immigrants.

The main explanatory variable in our study is the sex ratio in 1929, defined as the ratio of single males to single females for a given age range, ethnicity, and county of residence. We coded individuals as single in 1929 if their marital status in the 1930 census was never married or if they were married in 1930 and their first marriage occurred between 1929 and 1930. Our main analyses adopt a relatively strict interpretation of the age structure of the potential mates’ market, but we later evaluate the impact of relaxing certain of these restrictions. On average, males in our sample marry females that are younger by 4 years (the median is 3 years), which is similar to marital age gaps found in other studies. However, there is considerable diversity in these marital age gaps across immigrant groups. The median age gap between spouses was five years for Italian couples (largest), two years for Irish couples (smallest), four years for Polish and Russian couples, and three years for American, English, and German couples.

In addition to assuming an average age gap between spouses, our measure of the ethnic sex ratio (ESR) allows for both younger and older cohorts to be included in one’s potential marriage market. In our main models, we allowed for an age range of six years in one’s ethnic marriage market. Our ESR indicator is,

(1) $$ESR_{i,e,c}=\frac{\sum_{j=i}^{i+6}{Μ}_{j,e,c}}{\sum_{k=i-3}^{i+3}F_{k,e,c}}$$

where *M_j,e,c_* = # Single males in the age range *j* from ethnic group *e* and county *c*. *F_k,e,c_* = # Single females in the age range *k* from ethnic group *e* and county *c*.

Table 1 describes the variation in the distribution of the sex ratio by ethnicity in our 1930 sample of immigrants as well as for white natives for purposes of comparison. Means and standard deviations are calculated after removing cases with extreme sex ratios from the sample. Whereas the mean sex ratios of natives and Irish are around 1, the mean values for other ethnic groups are higher. Italians have the highest mean of sex ratios – 1.72, indicating that average marriage markets had 72% more males than females. In addition, the Italians also have the second highest variation and the highest percentage of extreme sex ratios that were eliminated from the sample. The table also presents the number of counties and marriage markets included in our sample and the percentage of cases that were excluded due to extreme marriage market squeeze. The data suggest that later migration streams settled in fewer counties than those who arrived earlier and had higher share of marriage markets with extreme sex ratio.

Since mate selection is modeled in terms of individuals competing for potential partners, we also control for the other ethnic groups’ sex ratio (OSR) within the same marriage market. This means that even if the ESR for German males at age 25 in county *x* is balanced, a shortage of females suitable for 25-year-old males of other ethnicities may mean greater competition for German males. Thus, the exogamy rate of German females in this case depends on the OSR as well on the ESR. The OSR is calculated as the percentage of males in ethnic groups other than the focal individual’s ethnic group (including natives) by age group and county:

(2) $$OSR_{i,o,c}=\frac{\sum_{j=i}^{i+6}{Μ}_{j,o,c}}{\sum_{k=i-3}^{i+3}F_{k,o,c}+\sum_{j=i}^{i+6}{Μ}_{j,o,c}}$$

where *M_j,o,c_* = # Single males from all other ethnic groups *o* in age range *j* from county *c*. *F_j,o,c_* = # Single females from all other ethnic groups *o* in age range *k* from county *c*.

Our models include the proportion of one’s ethnic group in the county (Blau, Blum, and Schwartz 1982; Choi and Tienda 2017), age, age at marriage, literacy as a proxy for education, urban residence, ethnicity, and immigrant generation. Table 2 shows summary statistics for the variables used in our analysis.

Our main analysis includes four separate models: three linear probability models (LPM) with heteroskedastic corrections to the standard errors and a fourth model based on the same specification as the third but also including county fixed-effects (FE) with robust standard errors. The county FE isolates the causal effect of the county level sex ratios while controlling for unobserved differences across counties, such as labor market conditions and complex histories of racial and ethnic discrimination by natives. The LPM and LPM-FE specifications allow us to focus on how the ethnic marriage squeeze affects the probability of intermarriage, while our sample size, use of robust standard errors, and extensive robustness checks ensure that our results are not affected by this modeling strategy.^3^

In the final set of models, we estimate individual marriage choices using multinomial logit regression with three outcomes: remain single, in-marriage, and out-marriage. The multinomial logit model estimates the relative effect of sex ratio on exogamy when we take into account remaining unmarried, the alternative response to the ethnic marriage squeeze.^4^

## 4. Results

Descriptive statistics on first and second-generation immigrant marriage patterns are shown in Table 3 and highlight the diversity by sex, ethnicity, and immigrant’s generation. Gender differences are prominent across groups: Among English immigrants, for example, the percentage of marriage is quite similar for males and females. In other groups, such as the Italians, the percentage of married females is much higher. These results match the age composition of the subsample of those who had never been married, where we find that the new immigrant groups are younger on average than the old immigrant groups (not shown). In terms of intermarriage, male rates are generally higher, although females marry out more among Irish and Polish immigrants.

Differences also emerge with respect to duration of exposure to the United States. As expected, intermarriage was more common among the older origin groups. In addition, the level of exogamy increased in the second-generation across all the ethnic groups for both sexes. For example, while 92% of the second-generation immigrant males from England married a partner without an English background, only 12% of the first-generation immigrant females from Italy married males from a different ethnic group.

Our main analysis follows in Table 4, where we present linear probability and fixed-effects models to examine the impact of ESR on the dichotomous outcome variable exogamy. The model sequence begins with a baseline (Model 1), which includes control variables but excludes the effect of sex ratios. The second model (Model 2) introduces ESR and its interaction with sex to show how ESR affects males and females differently. The third specification (Model 3) includes both ESR and OSR (with interactions) to estimate the impact of sex ratios within one’s ethnicity as well as outside one’s ethnic group. Model 4 uses county FE allowing us to control for differences across counties in their underlying and unobserved (fixed) differences. While most of our attention is focused on Model 4, the county FE model, we also discuss the coefficients from the other models.

Beginning with Model 1, we find that age has a negative effect on exogamy for the newly married, a signal of increasing flexibility in patterns of marriage for new cohorts in our data. Literacy, our proxy for education, is positively associated with exogamous marriage and urban residence has a negative effect on intermarriage.

Estimates show that community size is negatively and significantly associated with exogamy. Thus, every 10% increase (in absolute terms) in the proportion of the ethnic group within the county reduces the probability of exogamous marriage by over 11%. Inclusion of the control for community size helps to ensure that our subsequent sex ratio variables are capturing dimensions of the population structure that are beyond the gross effect of community size.

The ethnicity-generation indicators show that later streams of migrants (Italians, Polish, and Russians) exhibit lower probabilities of exogamous marriage relative to first-generation English immigrants. The estimates also show an increase in the probability of marrying out of one’s group when we compare second-generation immigrants to first-generation immigrants from within the same ethnic category (all contrasts are significant).

In Model 2, ESR is added along with an interaction between the ESR and the sex dummy. As expected (Hypothesis 1), ESR has an insignificant and negative effect on the probability of exogamous marriage for females and a positive significant effect on males. This means that when the ratio of males in the ethnic community increases, the probability of exogamy increases for males. The effect for females however is weak and nonsignificant. When controlling for OSR in Model 3, the effect of ESR on exogamy strengthen for both sexes and helps with the interpretation of the findings. In the county FE Model this trend continues.

Table 4 indicates that the impact of ESR by sex differs in magnitude. In quantitative terms, based on Model 3, decreasing ESR by 10% from a balanced level (ESR = 1.0) to an ESR of 0.9 is associated with a 0.11% decrease in the probability of females to marry out of their ethnic group. The effect of the ethnic marriage squeeze is qualitatively different and slightly larger for males: increasing ESR by 10% (from 1 to 1.1) leads to a 0.14 increase in the probability of exogamous marriage. Thus, while exogamy for both males and females is affected by sex ratios, males’ out-marriage is more strongly determined by the prevailing ESR (Hypothesis 2).

As expected in Hypothesis 3, OSR has a positive effect for females and a negative effect for males (Model 3). In Model 4, after including county fixed effects, the effect of OSR on exogamy for females is no longer statistically significant. For males, an increase of 10% in the number of males in other ethnic groups (in absolute term) decreases the probability for males to marry out by 1.1%.

**Figure 1:**
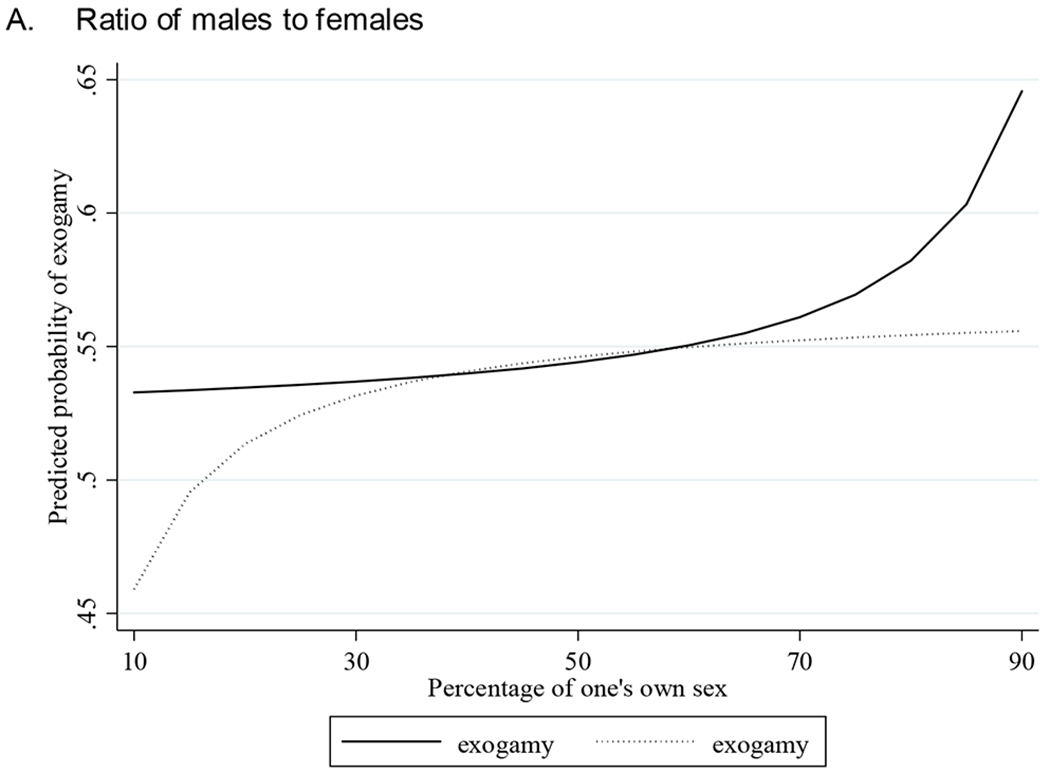

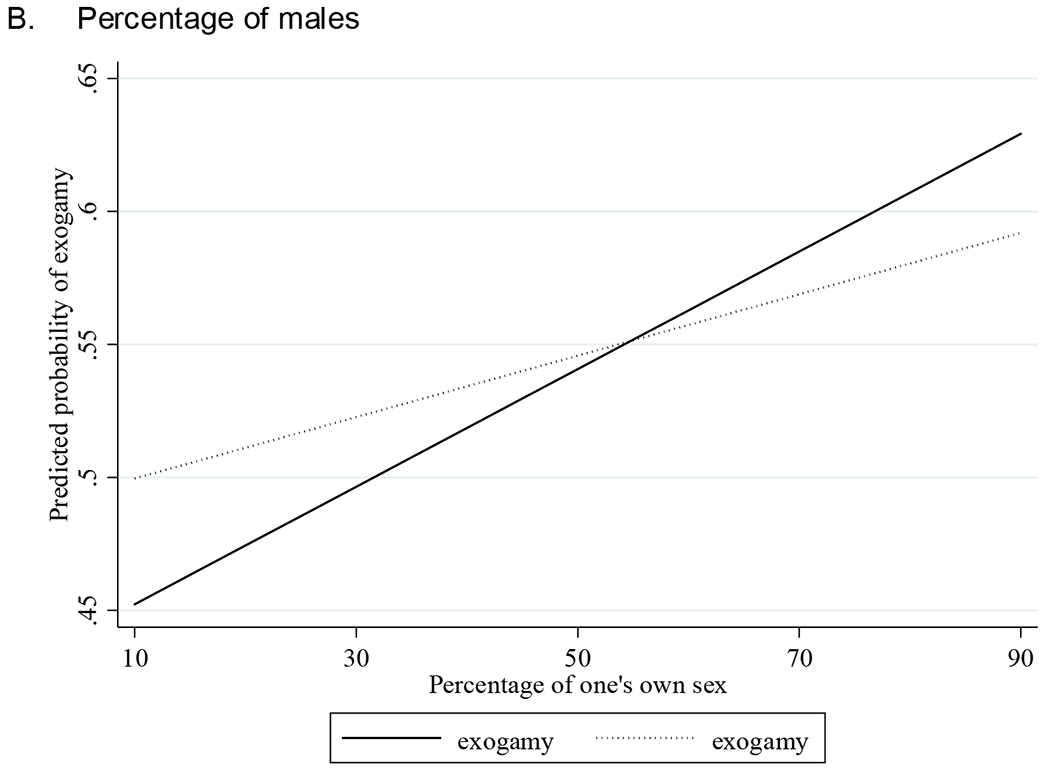

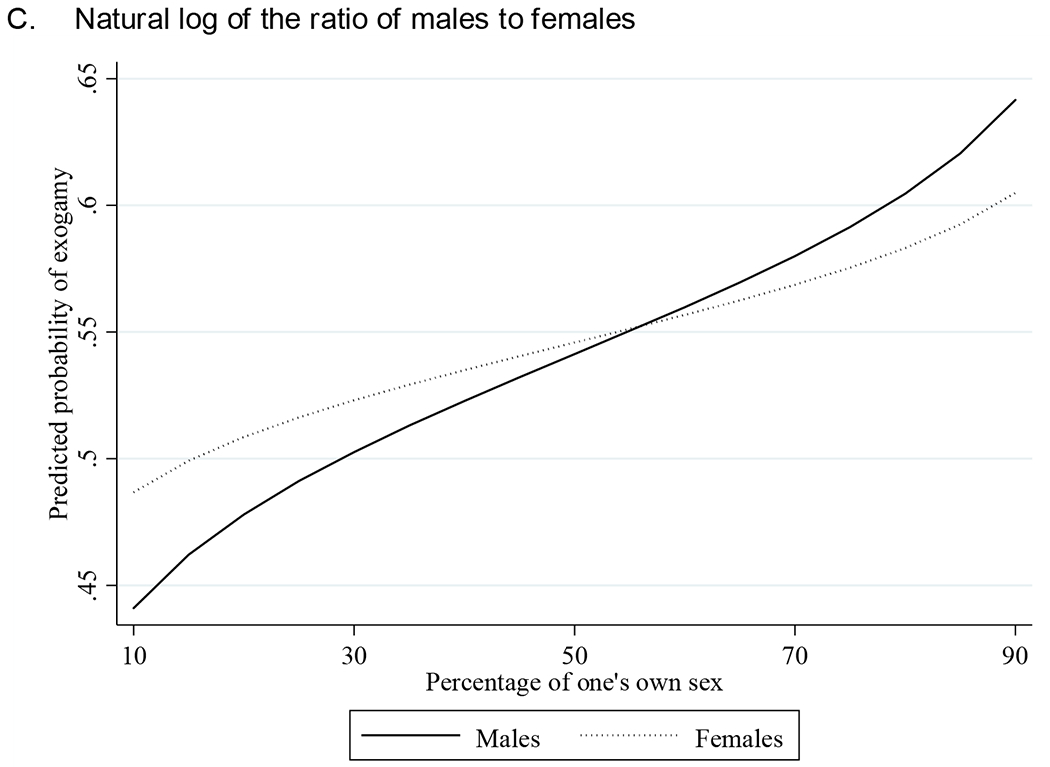
Predicted probabilities of exogamy by different measurements of ethnic sex ratio, sex, and percentage of one’s own sex in the ethnic marriage market (based on Model 4 in Table 4) *Source*: IPUMS, 1930 US census full count (Ruggles et al. 2018). *Note*: All other covariates are at their mean.

The predicted probabilities of exogamous marriage are plotted in Figure 1 for a range of ethnic sex ratios for males and females (all other covariates fixed at their averages or proportions). In order to test the robustness of our findings, we present the predicted probabilities that have been calculated using three different measurements of ethnic sex ratio. Plot A present the predicted probabilities of exogamy using the original ESR variable; Plot B uses the percentage of males in the marriage market; and Plot C uses the natural log of ESR. The x-axis’ values are the percentage of one’s own sex in the local marriage market.

Based on our county FE models, under equivalent degrees of marriage market constraints, the predicted probability of marrying out is higher for males in all three cases when experiencing a shortcut of potential partners. In addition, males’ sex ratio effects are stronger across all plots, as reflected by the steeper slopes of males.

In focusing on measuring how ESR affects ethnic intermarriage, our analysis has ignored the possibility that individuals chose to remain single when experiencing ethnic marriage squeeze. Our last set of main models (Table 5) expands the choices faced by also including nonmarriage as an outcome. We use a multinomial logit model with three categories: never married; endogamous marriage (baseline category); and exogamous marriage. Because age might have a strong effect on the log-odds of remaining unmarried versus endogamous marriage, we constrained the model to a sample of individuals who were 25 or older. As seen in Table 5, an increase in ESR decreases the log-odds for remaining unmarried versus endogamous marriage for females and increases it for males. A similar effect is seen in the log-odds for exogamous marriage versus endogamous marriage. Our results also suggest that, for males, ESR has a similar effect on remaining single and exogamous marriage compared to endogamous marriage. For females, the effect of ESR on remaining single compared to endogamous marriage is stronger and statistically different than the relative effect on exogamous marriage.

**Figure 2:**
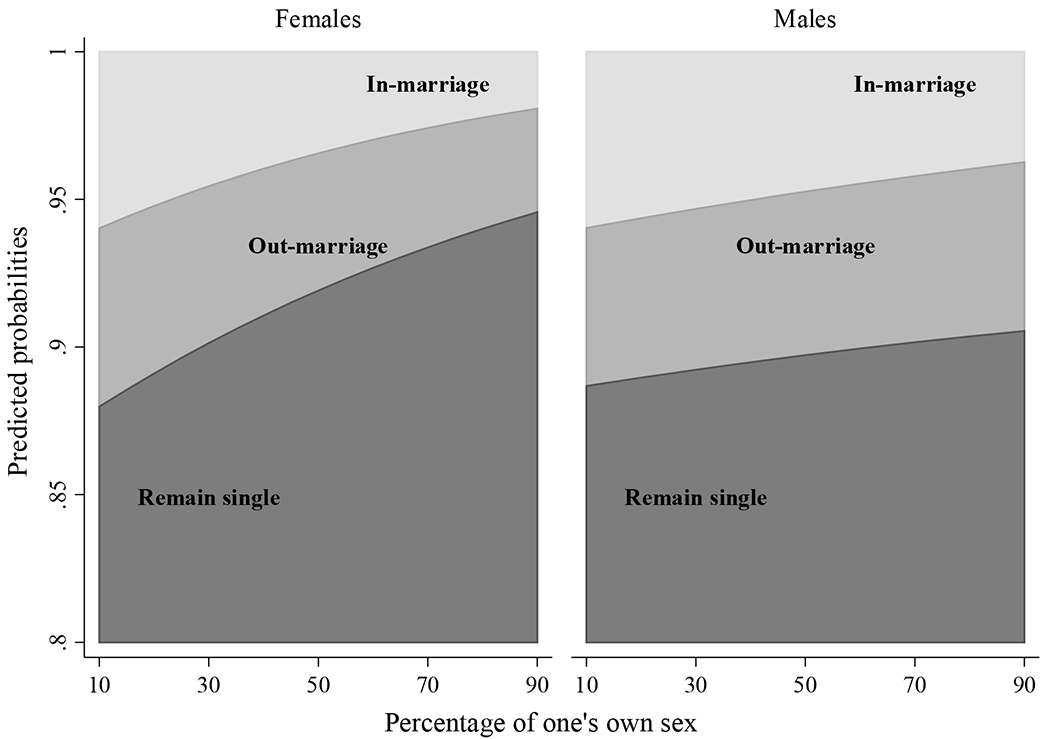
Predicted probabilities of remaining single, endogamous marriage, and exogamous marriage by sex and percentage of one’s own sex in the ethnic marriage market (based on Table 5) *Source*: IPUMS, 1930 US census full count (Ruggles et al. 2018). *Notes*: The results are based on a model using the percentage of males. Similar results were found using ESR as can be seen in Table 5. All other covariates are at their mean.

To better understand the effect of sex ratios on each outcome by sex we present the predicted probabilities of remaining single, endogamous marriage, and exogamous marriage for males and females by the percentage of their own sex in the ethnic marriage market (Figure 2). The predicted probabilities are based on a similar model to that in Table 5 but with percentage of males rather than ESR. The findings using the two measurements are qualitatively similar, which increases our confidence in the results. Overall, a shortage in potential partners increases the likelihood of both categories relative to endogamous marriage. For both sexes the effect of ESR on remaining single compared to endogamous marriage is higher than the effect of sex ratio on exogamous marriage compared to endogamous marriage. However, the difference between the slopes is more salient for females. While males were almost equally likely to remain single or to marry out of their ethnic groups when experiencing a shortage of potential partners, females were more likely to remain single than marry out in this scenario. Thus, while sex ratios accelerate the process of assimilation through intermarriage, the process is also determined by individual choices to remain unmarried. Focusing solely on intermarriage ignores part of the range of opportunities open to individuals in the market. The act of nonmarriage, either within or outside of one’s group, is likely very selective though producing substantial individual, social, and demographic consequences.

## 5. Robustness checks

While our findings point to clear and unambiguous impacts of ethnic sex ratios on the probability of exogamy for immigrants at the beginning of the 20^th^ century, we assess here the consequences of certain sample restrictions and measurement decisions. In Table 6 we present fixed-effects models for four separate variations on our main model (Model 4 in Table 4). All models in Table 6 include the same control variables and restrictions as those used earlier, unless otherwise noted, although only the main variables of interest are presented.

A first limitation of the study is that census data do not enable us to identify third generation immigrants. Therefore, while immigrants in our analysis may be classified as marrying natives (52.6% of the exogamous marriages in our data are between an immigrant and an American-born spouse), they may actually be marrying third (or more) generation immigrants from their own ethnic group (Kalmijn and van Tubergen 2010). While such unions might be rightfully classified as exogamous, it would be wise to assess the importance of this assignment. Although we cannot distinguish the American-born spouses that are third generational immigrants, we can test the robustness of our findings by excluding cases of immigrants who married so-called natives and classify exogamous marriages more narrowly as those between first- or second-generation immigrants from *different* ethnic groups (Model 1). The estimated impact of ESR remains very close to our earlier estimates and a larger difference was found in the impact of OSR.

A second concern is whether our decision to define ethnicity by the country of origin of *both* parents mattered. This approach led us to exclude individuals with immigrant parents from two different origin countries. This may introduce a bias if individuals who grew up in ethnically mixed families themselves have a higher tendency for intermarriage relative to those born to endogamous marriages (McCaa 1993). Model 2 reexamines our findings by changing the definition of ethnicity to rely solely on fathers’ ethnicity (Wildsmith, Gutmann, and Gratton 2003). This more relaxed interpretation of ethnicity means our sample also contains the offspring of exogamous marriages. As seen, our results for ESR remain consistent with earlier findings. The effect of OSR for males is similar in both models, but the direction for female has changed.

Similarly, defining ethnicity by national origin might identify inner-ethnic marriages of individuals from the same ethnic group that were born in different national states as exogamous marriages. To test whether our results are affected by our decision to operationalize ethnicity by country of birth, we reexamine our findings using a classification based on both place of birth and mother tongue (Pagnini and Morgan 1990). Model 3 is based on a sample of five ethnic groups: British, for immigrants from England, Scotland, and Wales; German, for immigrants from Germany or Poland if their mother tongue is German; Irish and Italian for immigrants from Ireland and Italy respectively; and Polish, for immigrants from eastern and central Europe who speak Polish. Although the sample and the ethnic groups are quite different, the results are qualitatively similar: women’s mate selections are less affected by ESR and OSR than men’s.

A fourth concern is whether our results are generalized due to the restriction of the sample to marriage markets that include at least 10% of both sexes. Some immigrant communities were extremely male dominant in 1930 and our results might ignore important marriage patterns in these markets. Model 4 includes individuals from all marriage markets. Since some of these markets include only males or only females, we cannot use Equation 1 as our sex ratio measurement. We use instead the percentage of males in the marriage market. The results are qualitatively similar to our main findings: the association between sex ratios and intermarriage is weaker for females compared to males. Overall, the effects are slightly stronger, most likely due to the inclusion of communities with extreme sex ratios.

Another concern is due to uncertainty about the appropriate age window for defining the marriage market of potential spouses and to what extent this might vary across ethnic groups. Model 5 presents an alternative specification where both ESR and OSR are based on age windows that allow us to capture over 90% of marriages (ranging from five years in favor of females to 11 years in favor of males). The method used in our main analysis limits us to capture about half of marriages. This additional model using a less restrictive age window indicates that the wider window for ESR has little impact on our substantive findings.

Our last model uses state-level analysis rather than county level. The predicted effect of ESR in the state level is no longer significant for females. For both sexes the effect of OSR is not statistically significant. To sum up, Table 6 indicates that our findings on the stronger ESR effect shown by males relative to females is robust to various sample definitions and operational measurements when using county level. The results are less robust when using state-level measurements. We also test our model using a city-level analysis including the ten most populated cities in terms of population in 1930 (not shown). The sex ratios in this sample are substantially more balanced than in the analyses by counties and states. Although the qualitative results using city level were the same as using county level, the gender differences were weaker. It is possible that the effects of ESR on intermarriage may work differently in urban and rural areas and future analysis should focus on the differences between the urban and the rural population.

## 6. Conclusion

Our study aims to shed light on if and how structural demographic forces helped drive patterns of exogamy and hence assimilation. Scholars of assimilation often point to intermarriage as one of its key stages. Through intermarriage, the boundaries between segments in society are blurred and the offspring of these unions are raised in cultural settings closer to the mainstream. From this perspective, increasing exogamy in America at the beginning of the 20^th^ century is crucial to this unique period in American history. Driven by the sex distributions of both international and domestic migration, sex ratios frequently became skewed and pushed individuals to shift their marital expectations. Even though ethnic community and ethnic identity were central in the daily lives of immigrants, mixed marriages existed, and our findings indicate that the shortage of potential spouses from the same ethnic background played an important role in stirring this process.

While previous studies have noted a relationship between sex ratios and marital assimilation, the gender differences in this association are understudied. Our analysis of the 1930 US census examines whether there are differences between males and females in the relationship between sex ratio and exogamy.

We find that males living in counties facing a shortage of potential spouses from within their own ethnic group were more likely to marry out of their group. Females during this period also responded to a shortage of eligible partners from their own group by marrying out, although not as readily as males.

The gendered nature of the growth in exogamy and in how sex ratios help to drive this process is not terribly surprising considering the role of women in transmitting ethnic identity and their lack of economic and legal resources. However, by 1930, 54.9% of male immigrants and 54.4% of female immigrants were marrying out of their ethnic group. The similar rate of intermarriage among males and female immigrants suggests that factors other than unbalanced sex ratio had a strong effect on women’s intermarriage.

Since intermarriage depends not just on the individual’s demands, but also on the supply of potential spouses from outside of one’s group, we also explored the effect of sex ratios of the other ethnic groups on exogamous marriage. Again, we found interesting differences by sex. While increased competition from other ethnic groups in the marriage market had a strong negative effect on male probabilities of intermarriage, the effect was insignificant for females. These findings raise the need to study mate selection as a process that is concurrently affected by both the demand for out-marriage and the pool of potential partners.

A limitation in our use of the 1930 data is that the 1930 census did not include a question on the number of previous marriages. Though our sample is restricted to individuals who married for the first time in the year before the survey and are, therefore, likely to be in their first marriage, we do not have estimates on exogamy in first versus second or higher marriage orders. The 1% sample of the 1940 census includes a question on times married. Using this dataset, we found that 17% of those in their first marriage and 17.7% of those in their second or higher marriage were in exogamous marriages.

The effect of the ethnic marriage squeeze contributes to our understanding of the forces driving assimilation. While many factors have contributed to patterns of assimilation, our findings emphasize the substantial role played by demographic structure and add fuel to the debate on assimilation. Even if education, social capital, and desegregation were all important processes in stimulating assimilation, the demographic structure of the population of immigrants, across counties in the United States, also played a key role. In this spirit, one of the contributions of this study is the inclusion of those remaining unmarried as an alternative to exogamy in response to the shortage of potential mates. In this approach, marital assimilation is referred to as a process that can be both accelerated and decelerated by unbalanced marriage markets. Interestingly, we find that for females the effect of sex ratios on remaining single versus endogenous marriage is stronger than the effect of sex ratios on exogamous marriage versus endogenous marriage. This suggests that women’s degree of assimilation was impeded by their choice to remain single in response to a shortage of potential males from their ethnic group. More work is needed to further elucidate these different responses to the ethnic marriage squeeze and to understand whether individuals from certain groups were more likely to opt for out-marriage while others tended to remain unmarried.

Our findings also suggest a broader need to comprehend whether this era was truly exceptional. Would our findings have been the same in different times? Might they explain patterns of assimilation for subsequent generations of immigrants? We argue that several factors highlight the uniqueness of this period. First, ethnicity remained so critical in this juncture of American society, making it particularly costly for individuals to marry out. Second, sex ratios may have been more unbalanced in 1930 than in later periods. Finally, gender differences may no longer be as large in later migration streams as women became more integrated into the labor market and less constrained in both their migration and marital choices over time.

## Figures and Tables

**Table 1:** Mean and standard deviation of ethnic sex ratios by age and county for the six largest immigration groups (in comparison to natives)

			*Cases excluded by extreme ESRs*		*Number of counties and marriage markets after excluding extreme ESRs*	
	Mean	SD	% ESR<0.11	% ESR>9	Counties	Marriage markets
American	1.00	0.45	<0.001%	0.135%	3,074	91,577
English	1.08	0.63	0.625%	1.657%	1,950	25,316
German	1.26	0.63	0.099%	0.744%	2,335	45,684
Irish	0.97	0.53	0.171%	0.828%	1,804	28,262
Italian	1.72	1.47	0.099%	7.094%	1,088	10,478
Polish	1.64	1.49	0.177%	3.630%	1,032	11,354
Russian	1.29	1.16	0.349%	4.630%	1,258	11,491

*Source*: IPUMS, 1930 US census full count (Ruggles et al. 2018).

**Table 2:** Descriptive statistics of the variables in the sample

	N	Mean	SD	Min	Max
**Dependent variable:**					
Exogamy		0.547			
Married between 1929 and 1930		0.107			
**Independent variables:**					
Ethnic sex ratio		1.275	0.970	0.111	9
Others sex ratio		0.514	0.067	0.165	1
Community size		0.094	0.065	0.001	0.698
Age		30.918	8.752	20	53
Age at marriage		26.613	5.896	19	53
Male		0.521			
Urban		0.823			
Literate		0.981			
Ethnicity:					
English	**251,703**				
First generation	83,399				
Second generation	168,304				
German	**865,108**				
First generation	178,546				
Second generation	686,562				
Irish	**684,564**				
First generation	179,716				
Second generation	504,848				
Italian	**381,764**				
First generation	151,184				
Second generation	230,580				
Polish	**267,267**				
First generation	86,022				
Second generation	181,245				
Russian	**187,338**				
First generation	48,724				
Second generation	138,614				

*Source*: IPUMS, 1930 US census full count (Ruggles et al. 2018).

**Table 3:** Probabilities of marriage between 1929 and 1930 and exogamous marriage (conditional on marriage) by ethnicity, sex, and immigrant generation

	Married between 1929 and 1930		Exogamy	
	1^st^ generation	2^nd^ generation	1^st^ generation	2^nd^ generation
English	0.119	0.117	0.774	0.913
Males	0.122	0.120	0.781	0.919
Females	0.117	0.114	0.765	0.908
German	0.139	0.109	0.293	0.756
Males	0.125	0.107	0.316	0.776
Females	0.161	0.110	0.263	0.736
Irish	0.071	0.059	0.279	0.756
Males	0.070	0.063	0.252	0.763
Females	0.071	0.055	0.304	0.748
Italian	0.154	0.129	0.170	0.309
Males	0.143	0.123	0.191	0.396
Females	0.189	0.133	0.119	0.243
Polish	0.099	0.126	0.331	0.420
Males	0.078	0.115	0.297	0.371
Females	0.159	0.135	0.378	0.456
Russian	0.127	0.143	0.442	0.520
Males	0.111	0.143	0.464	0.505
Females	0.162	0.143	0.409	0.532
American	0.147		0.202	
Males	0.145		0.182	
Females	0.149		0.220	

*Source*: IPUMS, 1930 US census full count (Ruggles et al. 2018).

**Table 4:** Linear probability and fixed-effects models for determinants of exogamy by marriage market characteristics at the county level, for ever-married immigrant population

	Model 1	Model 2	Model 3	Model 4
Age	−0.009 *** (0.002)	−0.010 *** (0.002)	−0.012 *** (0.002)	−0.007 ** (0.002)
Age squared	0.000 *** (0.000)	0.000 *** (0.000)	0.000 *** (0.000)	0.000 ** (0.000)
Age at marriage	0.002 (0.002)	0.002 (0.002)	0.002 (0.002)	0.002 (0.002)
Literate	0.130 *** (0.005)	0.133 *** (0.005)	0.131 *** (0.005)	0.125 *** (0.005)
Urban	−0.012 *** (0.002)	−0.009 *** (0.002)	−0.006 * (0.002)	0.006 (0.005)
Community size	−1.178 *** (0.013)	−1.164 *** (0.013)	−1.158 *** (0.013)	−1.191 *** (0.120)
English 2^nd^ Generation	0.139 *** (0.005)	0.139 *** (0.005)	0.139 *** (0.005)	0.128 *** (0.008)
German 1^st^ Generation	−0.393 *** (0.005)	−0.395 *** (0.005)	−0.395 *** (0.005)	−0.390 *** (0.023)
German 2^nd^ Generation	0.075 *** (0.005)	0.073 *** (0.005)	0.073 *** (0.005)	0.062 *** (0.013)
Irish 1^st^ Generation	−0.418 *** (0.006)	−0.418 *** (0.006)	−0.417 *** (0.006)	−0.400 *** (0.027)
Irish 2^nd^ Generation	0.036 *** (0.005)	0.036 *** (0.005)	0.037 *** (0.005)	0.045 ** (0.015)
Italian 1^st^ Generation	−0.494 *** (0.005)	−0.505 *** (0.005)	−0.501 *** (0.005)	−0.490 *** (0.015)
Italian 2^nd^ Generation	−0.365 *** (0.005)	−0.369 *** (0.005)	−0.367 *** (0.005)	−0.359 *** (0.015)
Polish 1^st^ Generation	−0.383 *** (0.007)	−0.392 *** (0.007)	−0.390 *** (0.007)	−0.372 *** (0.015)
Polish 2^nd^ Generation	−0.294 *** (0.005)	−0.297 *** (0.005)	−0.297 *** (0.005)	−0.281 *** (0.015)
Russian 1^st^ Generation	−0.301 *** (0.008)	−0.306 *** (0.008)	−0.305 *** (0.008)	−0.303 *** (0.017)
Russian 2^nd^ Generation	−0.208 *** (0.005)	−0.210 *** (0.005)	−0.209 *** (0.005)	−0.211 *** (0.015)
Sex (Male=1)	0.030 *** (0.002)	0.011 *** (0.003)	0.087 *** (0.015)	0.068 ** (0.020)
Ethnic sex ratio (ESR)		−0.000 (0.002)	−0.010 *** (0.002)	−0.011 ** (0.004)
Interaction: Sex*ESR		0.016 *** (0.002)	0.024 *** (0.002)	0.024 *** (0.004)
Others’ sex ratio (OSR)			0.242 *** (0.025)	0.022 (0.060)
Interaction: Sex*OSR			−0.162 *** (0.032)	−0.131 ** (0.040)
Intercept	0.792 *** (0.018)	0.800 *** (0.018)	0.724 *** (0.020)	0.764 *** (0.031)
R-square	0.255	0.255	0.255	0.254

N	281,722			

*Source*: IPUMS, 1930 US census full count (Ruggles et al. 2018).

*Notes*: Reference categories are Illiterate, rural, English 1^st^ Generation, and female.

* p <0.05 (two-tailed).

** p < 0.01 (two-tailed).

*** p <0.001 (two-tailed).

**Table 5:** Multinomial logit model for remaining single, endogamous marriage (baseline category), and exogamous marriage

	Remaining single	Exogamous Marriage
Ethnic sex ratio (ESR)	−0.141*** (0.008)	−0.067*** (0.012)
Interaction: Sex*ESR	0.185*** (0.009)	0.110*** (0.013)

R-square	0.091	
N	1,854,859	

*Source*: IPUMS, 1930 US census full count (Ruggles et al. 2018).

*Notes*: Endogamous marriage is the baseline category for the outcome variable. Female is the omitted category.

Age, age squared, age at marriage, literate, urban, community size, ethnicities, and OSR are also included in the models, but not shown.

* p <0.05 (two-tailed).

** p < 0.01 (two-tailed).

*** p <0.001 (two-tailed).

**Table 6:** Testing robustness of main model estimates (Table 4 Model 4: county fixed-effects models for determinants of exogamy) to alternative specifications

	Model 1	Model 2	Model 3	Model 4	Model 5	Model 6
	Sample includes married immigrants	Ethnicity defined by father	Ethnicity by mother tongue	All marriage markets	ESR with extended age gap	State-level FE
Sex (Male=1)	0.078*** (0.019)	0.066** (0.021)	0.084** (0.019)	−0.009 (0.022)	0.077** (0.025)	0.033 (0.033)
Ethnic sex ratio (ESR)	−0.013** (0.004)	−0.011** (0.004)	−0.004 (0.004)	−0.143*** (0.034)	−0.015** (0.005)	−0.002 (0.006)
Interaction: Sex*ESR	0.020*** (0.005)	0.025*** (0.004)	0.013*** (0.003)	0.361*** (0.024)	0.032*** (0.004)	0.016* (0.006)
Others’ sex ratio (OSR)	0.126 (0.067)	−0.020 (0.064)	0.005 (0.052)	0.093 (0.060)	0.032 (0.074)	−0.003 (0.086)
Interaction: Sex*OSR	−0.183*** (0.042)	−0.133** (0.040)	−0.118** (0.038)	−0.294*** (0.047)	−0.169*** (0.051)	−0.044 (0.066)

R-square	0.174	0.258	0.271	0.256	0.255	0.245
N	200,759	270,230	211,797	285,560	283,709	284,815

*Source*: IPUMS, 1930 US census full count (Ruggles et al. 2018).

*Notes*: Age, age squared, age at marriage, literate, urban, community size, and ethnicities are also included in the models, but not shown. Female is the omitted category.

* p <0.05 (two-tailed).

** p < 0.01 (two-tailed).

*** p <0.001 (two-tailed).

## Footnotes

3 Estimation using logistic regression produced results that are qualitatively similar to the linear probability models presented here.

4 We reestimated our main models using multinomial probit regression, which does not require the independence of irrelevant alternatives assumption. The multinomial probit and multinomial logit results were qualitatively similar but we present the logit models, which offer a more straightforward interpretation.
